# Clinical spectrum of chronic liver disease with final outcome in children at a tertiary centre: A single - centre study

**DOI:** 10.12669/pjms.37.3.3862

**Published:** 2021

**Authors:** Iqtadar Seerat, Eitzaz UD Din Khan, Muhammad Atique, Usman Iqbal Aujla

**Affiliations:** 1 Dr Iqtadar Seerat, MRCPCH, FRCPCH. Consultant and Head of Department of Paediatric Gastroenterology & Hepatology, Pakistan Kidney and Liver Institute & Research Centre, Lahore, Pakistan; 2 Dr. Eitzaz UD Din Khan, FCPS. Consultant & Head of Department of Anaesthesia, Pakistan Kidney and Liver Institute & Research Centre, Lahore, Pakistan; 3 Dr. Muhammad Atique, FCPS, FRCPath. Consultant and Head of Department of Pathology, Pakistan Kidney and Liver Institute & Research Centre, Lahore, Pakistan; 4 Dr. Usman Iqbal Aujla, Consultant Gastroenterologist , Pakistan Kidney and Liver Institute & Research Centre, Lahore, Pakistan

**Keywords:** Autoimmune hepatitis, Children, Chronic liver diseases

## Abstract

**Background and Objectives:**

Chronic liver disease (CLD) in children present a broad spectrum of symptoms. Limited resources in Paediatric Hepatology in developing countries like Pakistan present considerable challenges in investigating and treating children with chronic liver disease in a timely fashion. This study aimed to determine the spectrum and outcomes of CLD other than chronic hep B & C virus (HBV& HCV) liver disease in children.

**Methods:**

This retrospective descriptive study was conducted at the Paediatric Gastroenterology and Hepatology Department, Pakistan Kidney and Liver Institute and Research Centre in Lahore, Pakistan. The duration of the study was from August 2019 to January 2020. A total of 162 children of CLD were seen during this period of time. Of 162 there were 130 children with chronic HBV & HCV who were excluded from this study. 32 children aged 15 years or younger with chronic liver disease were included. The referrals were received from primary and secondary health care centres in different parts of the country. The data were collected from hospital electronic medical records database and then incorporated into a spreadsheet for analysis. The statistical analysis was performed by applying t-test with p value determined.

**Results:**

Of 32 children autoimmune hepatitis (n=11; 34.3%) was the most common cause for chronic liver disease referrals, followed by progressive familial intrahepatic cholestasis type-2, (n=7; 21.8%), post Kasai for biliary atresia, (n=4; 12.5%), glycogen storage disease type-1 (n=5; 15.6%), Wilson disease (n=3; 9.3%) and primary sclerosing cholangitis (n=2; 6.2%). The diagnosis was principally established with the assistance of liver ultrasound, liver biopsy, magnetic resonance cholangiopancreatography and genetic testing.

**Conclusion:**

Autoimmune hepatitis was the most common chronic liver disease. Our systematic approach, in addition to an extensive workup, helped us to diagnose and then initiate an appropriate treatment, which resulted in a more optimal outcome. Prompt referrals to tertiary centres are recommended where resources and expertise are available to reduce patient morbidity and mortality.

## INTRODUCTION

Children with chronic liver disease (CLD) could present with jaundice, itching, abdominal distension, pale stools and dark urine.^1^ Due to lack of resources and expertise in the field of Paediatric Hepatology especially in the developing world such as Pakistan, it is challenging to investigate and treat such diseases in children in a timely fashion. Hence, both morbidity and mortality increase in children. In this study our data revealed a variety of CLD with outcomes in children by evaluating a detailed history, clinical examination, appropriate investigations and treatment offered.^2^

## METHODS

This retrospective descriptive study with IRB no: PKLI-IRB/AP/021, dated 03/08/2020 was carried out at the Paediatric Gastroenterology and Hepatology Department, Pakistan Kidney and Liver Institute and Research Centre in Lahore, Pakistan. The duration of the study was from August 2019 to January 2020. The inclusion criteria were that children must be aged 15 years and younger with chronic liver disease of more than three months. Children older than age 15 years, with positive screening for HBV & HCV and acute liver diseases were excluded from the study population.

Of 162 there were 130 children with chronic HBV & HCV who were excluded from this study. Thirty two children aged 15 years or younger with chronic liver disease were recruited from primary and secondary health care centres in different provinces of Pakistan. Clinical notes were analysed in relation to clinical history, examination, investigations, and treatment to evaluate the outcome. The outcome was evaluated by comparing the improvement in liver function tests (LFTs), platelet count and INR (international normalised ratio) pre and post treatment. The data were collected from the electronic medical records database and incorporated into a spreadsheet for analysis. The statistical analysis was carried out by applying t test with p value determined.

## RESULTS

Of 32 children, 11 (34.3%) were diagnosed with Autoimmune Hepatitis, seven (21.8%) with Progressive Familial Intrahepatic Cholestasis (PFIC) type-2, four (12.5%) with Post Kasai for biliary atresia (BA), five (15.6%) with glycogen storage disease (GSD) type-1, three (9.3%) with Wilson Disease (WD) and two (6.2%) with Primary Sclerosing Cholangitis (PSC).

We classified the study population into three age groups: children aged one to five years (n=15), children aged five to 10 years (n=1), and children aged 10 to 15 years (n=16). Male-to-female ratio was 1.7:1. The overall study population mean age and standard deviation (SD) was 7.8±1.54. We further categorized mean age for each chronic liver disease. The mean age and SD for children with autoimmune hepatitis was (11.8±1.72), children with PFIC type-2 had a mean age and SD of (2.64±0.89), GSD type-1 children had a mean age and SD of (4.8±3.11), WD children had a mean age and SD of (11.33±2.08), post Kasai for BA children had a mean age and SD of (1.75±0.95) and children of PSC had a mean age and SD of (11.50±0.707).

The most common clinical features for chronic liver disease were jaundice, itching and abdominal distension secondary to ascites and haematemesis as shown in Table-I.

**Table-I:** Comparison of symptoms of CLD.

Symptoms	Present study (n=32)	Hanif 4 (n=55)	Malik 5 (n=30)
Jaundice	27(84%)	27 (49%)	22 (73%)
Abdominal distension	32 (100%)	44 (80%)	9 (30%)
Itching	13 (40%)	-	-
Haematemesis	8 (25%)	23 (42%)	4 (13%)

In addition to the LFTs a variety of other investigations like liver U/S, magnetic resonance pancreatography (MRCP), liver biopsy and genetic testing were carried out to confirm CLD as shown in Table-II.

**Table-II:** Investigations for chronic liver disease.

Investigations	Chronic liver diseases
Hepatitis B and C screen	Chronic hepatitis B and C
Serum caeruloplasmin, copper, 24 urinary copper after penicillamine challenge,	Wilson disease
Slit lamp examination	Wilson disease
Serum anti ANA, SMA, LKMA	AIH
Alpha1 antitrypsin level & phenotype	Alpha 1 antitrypsin deficiency
Liver ultrasound	Helpful in chronic liver disease
MRCP	PSC, AIH/ ASC
Liver biopsy	GSD, AIH,PSC etc.
Genotyping	GSD, PFIC

ANA, antinuclear antibody; SMA, smooth muscle antibody; LKMA, liver kidney microsomal antibody; CLD, chronic liver disease; MRCP, magnetic resonance cholangiopancreatography; PSC, primary sclerosing cholangitis; AIH, autoimmune hepatitis; ASC, ascending cholangitis; GSD, glycogen storage disease; PFIC, progressive familial intrahepatic cholestasis.

Routine laboratory investigations (LFTs, platelet count and INR) in Table-III showed a considerable improvement in four diseases after treatment given for 3-6 months and the p value was found to be significant (<0.05) only for children of AIH and PSC. Children of PFIC type-2 and post Kasai for BA presented with cholestasis but eventually lost their follow up due to unknown reasons, hence their pre and post treatment comparison was not mentioned in Table-III.

**Table-III:** P-value for Pre/Post treatment laboratory investigations (LFTs, Platelet count and INR) for chronic liver diseases

Variables	Mean (Pre & Post treatment)	P-Value
Wilson disease	147.87	0.49
	77.53	
Glycogen storage disease	231.78	0.62
	192.32	
Primary sclerosing cholangitis	161.26	0.147
	89.25	
Autoimmune hepatitis	108.98	0.042
	62.30	

## DISCUSSION

Jaundice and abdominal distension secondary to ascites were the major presenting symptoms in our study, being 84% and 100% as opposed to 49% & 73% reported from Hanif et al. and Malik et al. as shown in Table-I. Itching was observed in 13% of our patients and not found in 2 comparable studies.^3^,^4^

We found the AIH type-1 (positive anti ANA) as a commonest cause of CLD, was diagnosed by combination of clinical picture, laboratory work up and typical liver histology in the absence of a particular cause.^5^ The study conducted by Hanif et al.^3^ also showed that in their group of children of CLD, viral hepatitis B followed by Wilson disease were the commonest diseases. Another study performed in Pakistan revealed the hepatitis C and glycogen storage disease were the top two diseases in the list of their group of children.^6^ We excluded children of viral hepatitis B & C in our study.

AIH was confirmed with help of routine deranged liver function tests followed by high IgG, positive antinuclear antibody, backed up with U/S guided liver biopsy showing typical features of portal fibrosis, bile duct damage and inflammatory infiltrate comprising mostly eosinophils.^7^ They take azathioprine and a small dose of steroids daily and are in remission after receiving steroids during induction phase. Cirrhosis is generally present in between 44% and 80% of children of AIH at the time of diagnosis.^3^,^8^ Eleven (100%) children in our study presented with decompensated liver cirrhosis in the form of jaundice and ascites. AIH is relatively more aggressive disease in children than adults and therefore not diagnosing the disease promptly had resulted in decompensated liver cirrhosis in all eleven patients.

PFIC is a group of rare disorders caused by defect in bile secretion usually present with intra-hepatic cholestasis in children. The approximate incidence is 1 in 50,000-100,000 births.^9^,^10^ Depending on clinical picture, laboratory tests, liver histology and genetic testing, these are broadly divided into PFIC type 1, 2 and 3. The defect is ABCB4 gene protein for PFIC 2. Serum GGT is normal in patients with PFIC1/2.^11^ We also found normal GGT level in our group of children. Liver transplant is required in majority of the cases as they end up in liver cirrhosis. In this study PFIC type-2 children presented with cholestasis and were subsequently confirmed with liver biopsy and genetic testing. They received good nutritional support, fat soluble vitamins, medium chain triglycerides and antipruritic medications.

BA is a slow inflammatory process causing destruction of the bile ducts. If left untreated, the disease can lead to liver failure. Unfortunately, in our children surgery was offered quite late and therefore they ended up in failed Kasai operation. Only effective treatments for BA are the Kasai operation followed by liver transplant. Kasai portoenterostomy before the age of 8 weeks increases the life span of children and may delay liver transplant for years to come.^12^

GSDs are inborn errors of metabolism with abnormal deposition or utilization of glycogen. Non adherence to strict diet remains a contributory factor for the development of liver complications, decreased bone mass and renal disease. Dietary restriction generally helps to control metabolic effects of disease but the ultimate treatment remains an LT.^13^ GSD type-1 children in our study remain on restricted diet and their growth have been satisfactory. It happened after diagnosing with routine laboratory tests and liver biopsy which showed ballooned hepatocytes with central nuclei and focal areas of portal inflammation supported by positive genetic testing.

WD is a disease of copper metabolism, caused by mutations in the *ATP7B* gene required for copper excretion into the bile with increasing accumulation of copper in the central nervous system, eyes, kidneys, and heart.^14^,^15^ In our group of children the 24 hour urine copper level was high after penicillamine challenge. The serum caeruloplasmin was low and they did not require further investigations like liver biopsy and genetic testing as the slit lamp examination of eyes showed KF rings.^16^ Patients improved with zinc, penicillamine, pyridoxine and fat soluble vitamins.

PSC is rare, with an incidence of 0.2 in 100000 children.^17^,^18^ It is a chronic cholestatic disease featured by narrowing and destruction of the intra-hepatic and extra-hepatic bile ducts. Two PSC cases were confirmed with after deranged liver functions, abnormal U/S liver, MRCP and liver biopsy. One child did not require liver biopsy as the MRCP was abnormal showing stricturing disease of extra-hepatic bile ducts involving right and common hepatic ducts. The MRCP of second child was normal but the liver biopsy confirmed it by showing onion skinning of intrahepatic bile ducts. In PSC ursodeoxycholic acid may play a role in normalising LFTs.^19^ In both patients, we managed to achieve normal LFTs by giving ursodeoxycholic acid (dose of 15 mg/kg/day) and supportive medications for six months.

### Limitations of the study:

The study was retrospective and comprised a relatively less number of patients. However, it was carried out at a single tertiary centre and hence we recommend a larger study to take place in Pakistan to define the pattern of chronic liver disease in children.

## CONCLUSION

Autoimmune hepatitis was the common ailment among children with chronic liver disease who were referred to our hospital. With help of appropriate investigations and treatment in a timely fashion we were able to improve the outcome, pending liver transplant theses children may need in the future.

### Authors’ Contributions:

**IS (Principal Author):** Responsible and accountable for the accuracy and integrity of the work.

**EDK and MA:** Data Collection.

**UA:** Study Design and data Interpretation.
